# Evaluation of the Elecsys syphilis immunoassay for routine screening of serum samples in China

**DOI:** 10.1038/s41598-017-10103-9

**Published:** 2017-08-25

**Authors:** Chuanmin Tao, Xiaoke Hao, Wei Xu, Jie Zhang, Shiyang Pan, Zhihua Tao, Xiaofei Li, Junmei Chen, Bingchang Zhang, Yurong Qiu, Yanan Wu, Qishui Ou, Xianzhang Huang, Lanlan Wang

**Affiliations:** 1 Department of Laboratory Medicine, West China Hospital, Sichuan University, No. 37 Guoxuexiang, Chengdu, 610041 China; 2 Department of Clinical Laboratory Medicine, Xijing Hospital, Fourth Military Medical University, No. 15 Changle West Road, Xi’an, 710032 China; 3 Department of of Clinical Laboratory, The First Hospital of Jilin University, No. 71 Xinmin street, Changchun, 130021 China; 4 Department of Laboratory Medicine, Peking University Third Hospital, No. 49 North Huayuan Road, Beijing, 100191 China; 5 Department of Laboratory Medicine, The First Affiliated Hospital of Nanjing Medical University, No. 155 Hanzhong Road, Nanjing, 210029 China; 6 Department of Clinical Laboratory, The Second Affiliated Hospital of Zhejiang University School of Medicine, No. 88 Jiefang Road, Hangzhou, 310009 China; 7 Department of Clinical Laboratory, The Third People’s Hospital of Kunming, No. 391 Wujing Road, Kunming, 650011 China; 8 Department of Clinical Laboratory Center, Beijing Youan Hospital, Capital Medical University, No. 8 Youan Menwai Xitoutiao, Beijing, 100069 China; 9 Department of Clinical Laboratory, Shandong Province Hospital, No. 324 JingWu WeiQi Road, Jinan, 250021 China; 10 Department of Clinical Laboratory, Nanfang Hospital, Southern Medical University, No. 1838 North Guangzhou Avenue, Guangzhou, 510515 China; 11 Department of Clinical Laboratory, Fujian Provincial Hospital, Provincial Clinical College, Fujian Medical University, No. 134 DongJie, Fuzhou, 350001 China; 12 Department of Laboratory Medicine, The First Affiliated Hospital of Fujian Medical University, No. 20 Chazhong Road, Fuzhou, 350005 China; 13 Department of Clinical Laboratory Medicine, The Second Affiliated Hospital of Guangzhou University of Chinese Medicine, No. 111 Dade Road, Guangzhou, 510120 China

## Abstract

We compared the performance of the Roche Diagnostics Elecsys immunoassay for the detection of *Treponema pallidum* specific antibodies in patient serum samples with that of the Abbott Laboratories Architect chemiluminescent microparticle immunoassay and the InTec and KHB enzyme-linked immunosorbent assays, which are commonly used in China. We tested 13,767 serum samples collected from 13 independent laboratories throughout China, which included samples from 999 previously confirmed syphilis cases and 158 ‘borderline’ samples previously identified using the Architect, InTec, and KHB tests. The Mikrogen Syphilis Immunoblot was used to confirm positive test results. The consistency between the four different assays was 100%. The sensitivity of Elecsys immunoassay was 100% versus 98.26% for Architect, 99.11% for InTec; and 98.56% for KHB. The specificity of the Elecsys immunoassay was 99.81% versus 99.74% for Architect; 99.93% versus 99.80% for InTec; and 99.85% versus 99.77% for KHB. For borderline samples, the Elecsys immunoassay yielded no false-negative results and fewer false-positive results, compared to the other tests. Considering the ease-of-use, automation, high speed, and high throughput capacity of the Elecsys assay, the higher sensitivity and specificity indicate it is superior for routine screening of serum samples for syphilis diagnosis.

## Introduction

In 2008, China had 278,215 officially reported cases of syphilis, which represented a three-fold increase, compared to the prevalence of syphilis in 2004, and an approximate ten-fold increase over the prevalence reported in the 1990s^1–3^. Guangdong Province, the largest province in southern China, reported more syphilis cases in 2008 than all of the countries in the European Union combined. Guangzhou City, the provincial economic center, was most affected, where 43.4 latent cases per 100,000 were reported in 2013 for all patient groups aged 20 years and older^4^ and 31 cases per 100,000 were reported the following year^5^. The control of *Treponema pallidum* transmission and infection is, therefore, a public health priority in China^4^.

Factors affecting the immunogenicity of *T. pallidum* include the relative scarcity of outer membrane proteins and antigenic variation of the putative surface-exposed TprK protein^6^. However, syphilis can be successfully treated, and early diagnosis is crucial to prevent transmission and avoiding delays in treatment^7, 8^. Testing for syphilis is common in antenatal care, screening of blood and organ donors, and surveillance of sexually transmitted infections^8–11^. However, there is no commonly accepted gold standard for syphilis diagnostic testing, and the algorithms used for initial screening and diagnosis confirmation vary between different countries^8–11^. The fastidious nature of *T. pallidum* prevents culturing using *in vitro* methods, and direct testing of swabs of primary lesions is often not possible because the short-lived lesions may occur internally. As a result, serologic testing is regarded as the preferred method for syphilis diagnosis and formmonitoring treatment outcomes^8^.

With the increased burden of syphilis and the dramatic increase in the number of patients requiring testing in China, a critical demand exists for a simple, accurate, rapid, and high-throughput test for *T. pallidum* detection. Currently, enzyme-linked immunosorbent assays (ELISA) are the most widely used methods for identifying antibodies to *T. pallidum* in both clinical and blood bank settings. These include the InTec (Xiamen, China)^12^ and KHB (Shanghai Kehua Bioengineering, Shanghai, China)^13^ double-antigen sandwich (DAGS) ELISA tests. These tests are, however, not high-throughput methods, and chemiluminescence-based automated assays offer advantages in terms of high-throughput capacity and sensitivity, which is important for early-stage syphilis cases^14^. The Architect syphilis TP system (Abbott Laboratories, Abbott Park, IL, USA) uses paramagnetic microparticle capture of *T. Pallidum* specific combined with chemiluminescent detection^15^. However, the Architect system is not fully automated. By contrast, the newly developed Elecsys syphilis assay (Roche Diagnostics, Indianapolis, IN, USA) is a fully automated electrochemiluminescence immunoassay that detects *T. pallidum* specific antibodies in human serum samples^16^.

We performed a multicenter study to compare the performance of the Elecsys immunoassay with that of the Architect, InTec, and KHB syphilis tests based on the analysis of serum samples collected at various locations throughout China. Our results indicated that the Elecsys immunoassay is superior to these other methods for the routine detection of *T. pallidum* in human blood samples.

## Materials and Methods

### Study design

This multicenter evaluation involved 13 independent laboratories in 10 different regions in China, including Beijing, Xi’an, Fuzhou, Jinan, Guangzhou, Chengdu, Kunming, Nanjing, Hangzhou, and Changchun. The main study objective was to compare the sensitivity and specificity of the Elecsys syphilis assay with that of the Architect, InTec, and KHB tests, which are commonly used in China. Our study was conducted in accordance with the Declaration of Helsinki with regard to ethical principles for research involving human subjects, and our study protocol was approved by the ethics committee of each participating institution (West China Hospital, Xijing Hospital, The First Hospital of Jilin University, Peking University Third Hospital, The First Affiliated Hospital of Nanjing Medical University, The Second Affiliated Hospital of Zhejiang University School of Medicine, The Third People’s Hospital of Kunming, Beijing Youan Hospital, Shandong Province Hospital, Nanfang Hospital, Fujian Provincial Hospital, The First Affiliated Hospital of Fujian Medical University, and The Second Affiliated Hospital of Guangzhou University of Chinese Medicine). All participants were 18 years of age or older, and written informed consent was obtained from each participant prior to our analysis.

### Samples

Samples from 999 patients with clinically and laboratory confirmed syphilis and 158 samples with borderline results for the Architect, InTec, and KHB tests (*n* = 158) were included in our analysis (Table 1). All of the samples (*n* = 13,767) were assigned an anonymous identifier prior to testing, and were screened in parallel. The samples comprised routine specimens that were collected from patients scheduled for surgery, persons at high risk of syphilis infection, pregnant women, and other types of common diagnostic requests.

**Table 1:** Study sites, methods, and samples for each sample group.

Study site	Assay methods	Samples Source		
		Confirmed positive* (*n* = 999)	Routine (*n* = 13,767)	Borderline (*n* = 158)
Beijing, Xi’an, Fuzhou (2 sites), Jinan, Guangzhou	Architect	355	6,004	72
Chengdu, Kunming, Nanjing, Hangzhou	InTec ELISA	391	4,820	71
Beijing, Changchun, Guangzhou	KHB ELISA	253	2,943	15
	Elecsys	999	13,767	158

Definiltion of abbreviations: ELISA = enzyme-linked immunosorbent assay.

*Samples from patients with a confirmed diagnosis of syphilis.

### Assays

**Figure 1:**
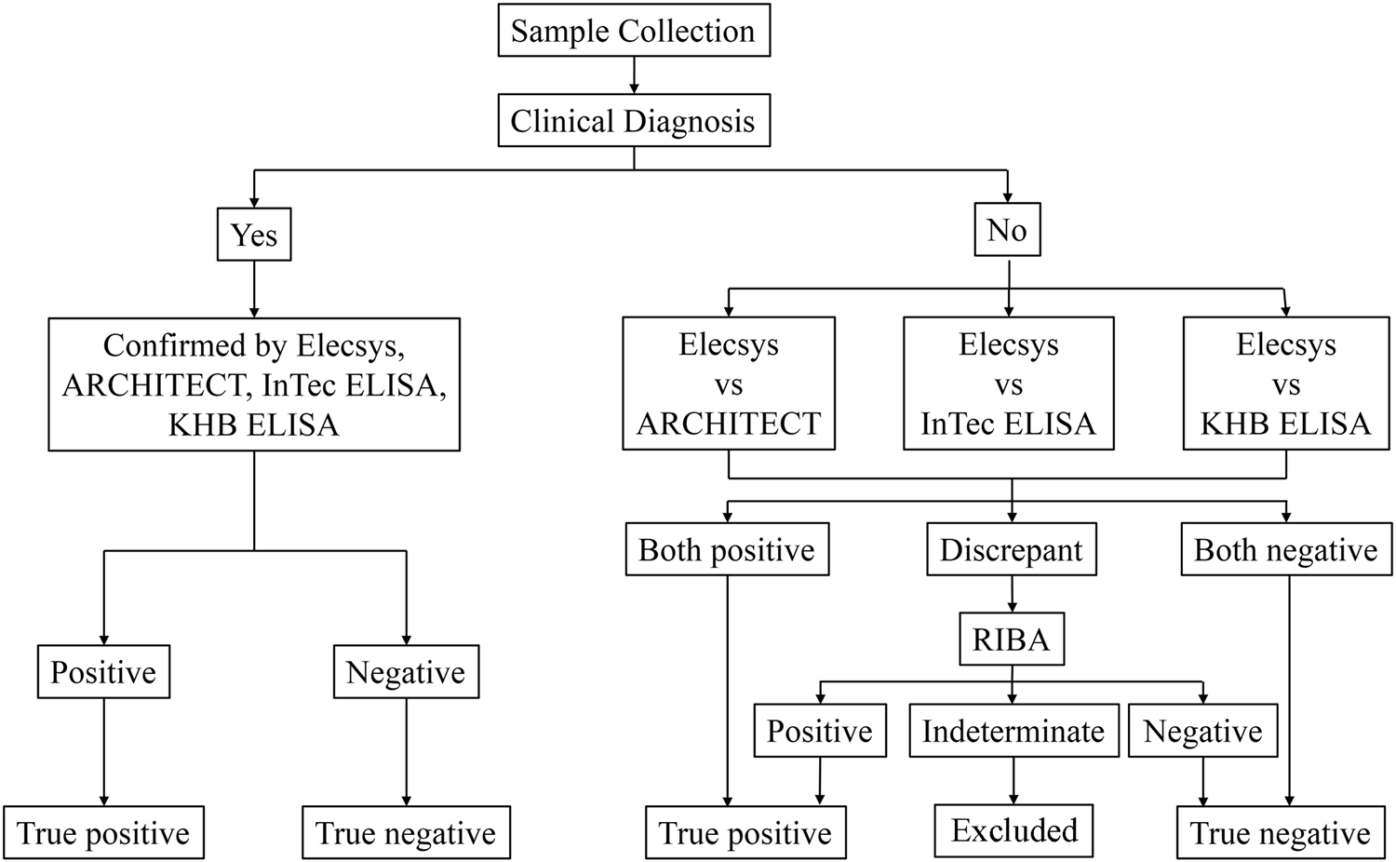
Testing algorithms for Treponema pallidum detection using the Elecsys, Architect, InTec, and KHB assays.

All samples were tested according to the manufacturer’s instructions for each assay. The manufacturers’ recommended cut-off indices (COIs) were <1.00 and ≥1.00 for negative and positive results, respectively. The borderline samples and all other samples for which the results between the different assays were inconsistent were further subjected to examination using the Mikrogen Syphilis Immunoblot (Mikrogen Diagnostik, Neuried, Germany) to confirm the presence of *T. pallidum* specific antibodies. Using this approach, the outcomes of the Elecsys, Architect, InTec, and KHB assays were categorized as positive, false-positive, negative, or false-negative according to the flow diagram in Fig. 1. Samples with an indeterminate result for immunoblotting were excluded from the interassay comparisons (Fig. 1).

### Elecsys Syphilis assay

The Elecsys Syphilis assay was performed using the Cobas e 601 platform (Roche Diagnostics). The automated Elecsys immunoassay simultaneously detects anti-treponemal IgG and IgM antibodies in serum or plasma. A 10-µL aliquot of each sample was incubated with a mixture of biotin- and ruthenium-labeled recombinant TpN15, TpN17, and TpN47 antigens to form a DAGS immune complex, after which streptavidin-coated paramagnetic microparticles were added. Following biotin-streptavidin binding, the immune complex attached to the microparticles was then magnetically sequestered on the electrode. Chemiluminescence was electrically induced and measured. The analyzer software automatically calculated the results, and the average testing duration was approximately 18 min.

### Architect Syphilis TP assay

The Architect syphilis TP system uses paramagnetic microparticles coated with recombinant TpN15, TpN17, and TpN47 to bind and purify IgG and IgM antibodies specific for *T. pallidum*. After incubating the microparticles with 30 µL of serum, murine anti-IgG/anti-IgM acridinium-labeled antibody conjugates were added, and chemiluminescence was measured. The average testing duration was approximately 29 min.

### InTec and KHB ELISA tests

Both of these tests are two-step DAGS ELISA tests in which the assay plates have been coated with the recombinant *T. pallidum* antigens, TpN15, TpN17, and TpN47. After incubating 100 µL of serum in each well, *T. pallidum* specific IgG and IgM antigen-antibody complexes were detected by the addition of horseradish-peroxidase-labeled TpN15, TpN17, and TpN47 antigens, which were visualized using 3,3′,5,5′-Tetramethylbenzidine (TMB) as the chromogenic substrate. The average testing duration was approximately 2 h.

### Mikrogen Syphilis Immunoblot

The recomLine Treponema IgG and IgM kits were used to confirm the detection of IgG and IgM antibodies against *T. pallidum* by immunoblotting (TPIB). Nitrocellulose membrane test strips containing the Tp47, TmpA, Tp257, Tp453, Tp17 and Tp15 antigens were incubated with 20-µL serum samples, which was followed by incubation with horseradish-peroxidase-labeled secondary antibodies specific for human IgG or IgM. The immune complexes were visualized as dark bands on the strips after the addition of TMB. The intensity of each antigen band was assessed by comparison with the intensity of the cut-off control band. A test has been considered as positive when at least two of six diagnostic bands, which corresponded to

TmpA, Tp47, Tp257, Tp453, Tp15 or Tp17 were recognized as reactive. Using initial serum incubation times of 1 or 3 h, the average testing duration was approximately 4 or 6 h.

## Results

### Interassay comparisons of consistency, sensitivity, and specificity

We first compared the sensitivities of the various assays for samples from the 999 previously confirmed syphilis cases according to disease stage (Table 2). No differences were observed in the results for these samples between the Elecsys, Architect, InTec, and KHB tests. A comparison of consistency between the results of the various tests for the routinely screened samples revealed no significant interassay variation (Table 3), with consistencies of >99.30% for each of the methods evaluated. The results of interassay comparisons for the routinely screened samples revealed sensitivities for the Elecsys test of 100% versus 98.26% for the Architect test, 99.11% for the InTec ELISA, and 98.56% for the KHB ELISA. The specificity of the Elecsys immunoassay was also higher than those of the Architect, InTec, and KHB tests (Table 4).

**Table 2:** Sensitivities of the various assays based on the analysis of 999 confirmed syphilis cases according to clinical stage.

Stage	Elecsys vs. Architect			Elecsys vs. InTec ELISA			Elecsys vs. KHB ELISA		
	N	Elecsys	Architect	N	Elecsys	InTec ELISA	N	Elecsys	KHB ELISA
I (primary)	20	100%	100%	44	100%	100%	2	100%	100%
II (secondary)	55	100%	100%	74	100%	100%	24	100%	100%
III (tertiary)	6	100%	100%	24	100%	100%	6	100%	100%
Latent	96	100%	100%	149	100%	100%	56	100%	100%
Unclassified	178	100%	100%	100	100%	100%	165	100%	100%
Total	355	100%	100%	391	100%	100%	253	100%	100%

Definition of abbreviations: ELISA = enzyme-linked immunosorbent assay.

**Table 3:** Consistencies of the various assays for routine screening 13,767 clinical samples.

Assay methods	Overall	Positive	Negative	Consistency	Kappa value	*P*-value
Architect	6,004	136 (2.27%)	5,868 (97.73%)	99.30	0.840	0.176
Elecsys	6,004	132 (2.20%)	5,872 (97.80%)			
InTec ELISA	4,820	232 (4.81%)	4,588 (95.19%)	99.65	0.961	0.730
Elecsys	4,820	231 (4.79%)	4,589 (95.21%)			
KHB ELISA	2,943	279 (9.48%)	2,664 (90.52%)	99.49	0.970	0.873
Elecsys	2,943	282 (9.58%)	2661 (90.42%)

**Table 4:** Specificities and sensitivities of the various assays for routine screening of 13,767 clinical samples.

	Elecsys vs. Architect		Elecsys vs. InTec ELISA		Elecsys vs. KHB ELISA	
	Elecsys	Architect	Elecsys	InTec ELISA	Elecsys	KHB ELISA
Total samples tested	6,004	6,004	4,820	4,820	2,943	2,943
Both assays positive	113	113	223	223	273	273
Both assays negative	5849	5849	4580	4580	2655	2655
Inconsistent results	42	42	17	17	15	15
Confirmed positive by TPIB	2	0	2	0	4	0
False-positive	11	15	3	9	4	6
No clear result by TPIB*	6	8	3	0	1	0
Confirmed Negative by TPIB	15	11	9	3	6	4
False-negative	0	2	0	2	0	4
No clear result by TPIB*	8	6	0	3	0	1
Sensitivity	100%	98.26%	100%	99.11%	100%	98.56%
(95% CI,2-sided)	(96.84–100%)	(93.86–99.79%)	(98.37–100%)	(96.83–99.89%)	(98.68–100%)	(96.34–99.61%)
Specificity	99.81%	99.74%	99.93%	99.80%	99.85%	99.77%
(95% CI,2-sided)	(99.67–99.91%)	(99.58–99.86%)	(99.81–100%)	(99.63–99.91%)	(99.62–99.96%)	(99.51–99.92%)
NPV	100%	99.97%	100%	99.96%	100%	99.85%
PPV	91.27%	88.28%	98.68%	96.12%	98.58%	97.85%

*Samples with indeterminate confirmation by the TPIB test were excluded from the evaluations of sensitivity and specificity.

Definition of abbreviations: CI = confidence interval; ELISA = enzyme-linked immunosorbent assay; NPV = negative predictive value; PPV = positive predictive value.

### Subgroup analysis of the borderline samples

The Elecsys syphilis assay was also assessed based on the analysis of 158 borderline samples previously identified using the Architect, InTec, and KHB tests (Table 5). The TPIB analysis revealed the following results: 28 false-positives among the 72 Architect samples versus 6 false-positives for the Elecsys assay using the same false-positive sample COI range as the Architect; 19 false-positives and 2 false-negatives among the 71 InTec ELISA samples versus 15 false-positives and no false-negatives for the Elecsys immunoassay with 5 samples in different false-positive COI ranges; and 3 false-positives among the 15 KHB ELISA samples versus 3 false-positives for the Elecsys immunoassay, 2 of which were misdiagnosed by both assays using the same COI range.

**Table 5:** Comparison of false-positive and false-negative results from 158 borderline samples between Elecsys, Architect, InTec ELISA and KHB ELISA assays with TPIB as the confirmation test.

Sample range	Samples	Architect, InTec, and KHB*								Elecsys							
		Positive	TPIB			Negative	TPIB			Positive	TPIB			Negative	TPIB		
			P	N	I		P	N	I		P	N	I		P	N	I
Architect (0.9 < COI < 5)																	
0.9 –<1.0	2	0	0	0	0	2	0	1	1	1	0	0	1	1	0	1	0
≥1.0 –<2.0	36	36	6	**18**	12	0	0	0	0	15	6	**6**	3	21	0	12	9
≥2.0 –<3.0	19	19	7	**8**	4	0	0	0	0	7	7	0	0	12	0	8	4
≥3.0 –<4.0	9	9	6	**2**	1	0	0	0	0	6	6	0	0	3	0	2	1
≥4.0 –<5.0	6	6	6	0	0	0	0	0	0	6	6	0	0	0	0	0	0
Total	72	70	**28 FP**			2	0 FN			35	**6 FP**			37	0 FN		
InTec ELISA (0.75 < COI < 3)																	
0.75 –<1.0	27	0	0	0	0	27	**2**	20	5	12	2	**5**	5	15	0	15	0
≥1.0 –<2.0	34	34	10	**15**	9	0	0	0	0	25	10	**7**	8	9	0	8	1
≥2.0 –<3.0	10	10	4	**4**	2	0	0	0	0	8	4	**3**	1	2	0	1	1
Total	71	44	**19 FP**			27	**2 FN**			45	**15 FP**			26	0 FN		
KHB ELISA (0.75 < COI < 3)																	
0.75 –<1.0	6	0	0	0	0	6	0	6	0	1	0	**1**	0	5	0	5	0
≥1.0 –<2.0	4	4	4	0	0	0	0	0	0	4	4	0	0	0	0	0	0
≥2.0 –<3.0	5	5	2	**3**	0	0	0	0	0	4	2	**2**	0	1	0	1	0
Total	15	9	**3 FP**			6	0 FN			9	**3 FP**			6	0 FN		

For the Architect, InTec, and KHB assays, a COI < 1.0 was considered to be negative and ≥1.0 considered positive.

Definition of abbreviations: COI = cut-off index; ELISA = enzyme-linked immunosorbent assay; FN = false-negative; FP = false-positive; P = positive; N = negative; I = indeterminate; TPIB = recombinant immunoblot assay.

## Discussion

The recent resurgence of syphilis has become a serious threat to public health worldwide, and is a particular problem in China. Its prevalence must be determined by comprehensive screening of blood donations, pregnant women, and organ transplantation donors. Therefore, an accurate, rapid, high-throughput laboratory method of *T. pallidum* detection is needed to aid in the diagnosis of syphilis.

Serologic tests for *T. pallidum* detection are classified as non-treponemal or treponemal assays. Treponemal tests detect *T. pallidum* specific antibodies^14, 17^. By contrast, non-treponemal tests, such as the Rapid Plasma Reagin (RPR) or Venereal Disease Research Laboratory (VDRL) tests, detect antibodies against host-cell lipids, such as cardiolipin and lecithin, which are released from cells upon *T. pallidum* infection. Non-treponemal tests can, however, produce false-positive results due to viral infections, pregnancy, and certain autoimmune disorders^18, 19^, and have limited sensitivity for primary, late, or latent stage syphilis cases^20^.

Current guidelines leave the choice of first-line laboratory testing for syphilis diagnosis to the discretion of the physician or clinical laboratory. The results of our study revealed that the Elecsys immunoassay demonstrated superior specificity and sensitivity for routine sample screening, compared to that of the Architect, InTec, and KHB tests (Table 4). In the analysis of the borderline samples, the Elecsys immunoassay produced no false-negatives and fewer false-positive results than the other tests (Table 5), with the Architect, InTec, and KHB tests producing at total of 50 false-positives and 2 false-negatives for the borderline samples, whereas the Elecsys test produced only 24 false-positives and no false-negatives. The use of the TpN15, TpN17, and TpN47 antigens in all of the assays evaluated suggest that the differences in the technologies used by the various tests contributed to the differences in the false-positive rates.

The fully automated Elecsys syphilis assay is well suited for providing results rapidly in high-volume clinical laboratories. With a sample assay volume of only 10 μL and an average testing duration of only 18 minutes, the Elecsys test delivers results faster than the Architect, InTec, and KHB tests. The specificity and sensitivity of the Elecsys syphilis assay were also superior to the Architect, InTec, and KHB tests, which indicates that it represents a good choice for routine first-line screening of blood samples for syphilis diagnosis in China. Given that confirmatory treponemal or non-treponemal tests are recommended for reverse algorithms, our results revealed that false-negatives can be completely eliminated by using the Elecsys immunoassay with the Architect, InTec, or KHB immunoassay.

In conclusion, based on its high levels of sensitivity and specificity for *T. pallidum* screening, the Elecsys syphilis assay represents an excellent first-line laboratory diagnostic test for syphilis diagnosis in China. For the routine screening of blood samples, it has the advantage of being fast, easy to use, and can be used in a high-throughput platform. However, like other treponemal assays, the Elecsys syphilis assay is suitable for the diagnosis of recently acquired and previously treated infections only. Its use in the diagnosis of advanced disease requires other laboratory results and clinical findings.
